# Camrelizumab as a novel third or post-third-line treatment strategy in small cell lung cancer: a retrospective study of 12 patients

**DOI:** 10.3389/fonc.2023.1180735

**Published:** 2023-07-04

**Authors:** Jizheng Tian, Lili Sui, Hong Wang, Xiaoyan Chen

**Affiliations:** Department of Oncology, Beijing Shunyi District Hospital, Shunyi Teaching Hospital of Capital Medical University, Beijing, China

**Keywords:** SCLC, camrelizumab, PD1 inhibitor, chemotherapy, case series

## Abstract

**Background:**

Small cell lung cancer (SCLC) constitutes 15% of all lung cancer cases, with a comparatively low survival rate. The advent of immune checkpoint inhibitors (ICIs) has provided new alternatives for treating SCLC. However, the effectiveness of camrelizumab in the treatment of SCLC remains unclear. This retrospective case series was designed to investigate the efficacy and safety of camrelizumab in SCLC patients.

**Methods:**

The study enrolled SCLC patients recorded as having received more than one cycle of camrelizumab in the electronic medical record system. Data related to clinical and survival times were collected and statistically analyzed.

**Results:**

From August 2019 to December 2021, the study enrolled 12 SCLC patients. The objective response rate was 41.7% (95% confidence interval [CI]: 15.2%–72.3%). The disease control rate was 83.3% (95% CI: 51.6%–97.9%). The median progression-free survival (PFS) for all patients was 4.0 months. Notably, the median PFS of patients in third- or post-third-line subgroups was 7 months (95% CI: 1.12–12.88 months). The median overall survival (OS) for all eligible patients was 10.0 months (95% CI: 7.35–12.65 months), with a 1-year survival rate of 25%. Notably, the OS of patients treated with third- or post-third-line therapy was 5–34 months, with a 1-year survival rate of 75%. The two most prevalent non-hematological adverse events associated with the immune response were pneumonitis (44.4%) and reactive cutaneous capillary endothelial proliferation (44.4%). One patient experienced an exacerbation of preexisting diabetes and reached grade 3 hyperglycemia. There were no grade 4/5 immune-related adverse events.

**Conclusion:**

This case series highlights the potential benefits and safety concerns of camrelizumab in SCLC patients. These findings suggest a possible strategy for third- and post-third-line treatments of SCLC. However, the conclusion is limited due to the study’s retrospective nature and small sample size. Therefore, large-scale randomized controlled studies are needed to determine its efficacy.

## 1 Introduction

Small cell lung cancer (SCLC) is a common malignancy with a low 2-year survival rate among all cancers (14%–15%) (1). In 2021, nearly 30,000 new cases of SCLC were diagnosed in the United States (2). For over 30 decades, etoposide plus platinum has been the preferred treatment and the cornerstone of therapy for SCLC patients (3). However, significantly reduced benefits for relapsed patients after first-line treatment were observed. The advent of immunotherapy has ushered in new hope for the therapy of SCLC.

Previous genomic research has demonstrated a high tumor mutation burden in SCLC (4) that is predicted to respond to immunotherapy. The Food and Drug Administration (FDA) has approved several immune checkpoint inhibitors (ICIs) for SCLC. One study confirmed that the cytotoxic T lymphocyte-associated antigen-4 inhibitor ipilimumab plus chemotherapy could significantly improve the median progression-free survival (PFS) (5). Four phase III studies, IMpower 133, CASPIAN, ASTRUM-005, and CAPSTONE-1, showed that programmed death 1 (PD-1)/programmed death ligand 1 (PD-L1) blockers plus chemotherapy are beneficial for untreated extensive-stage SCLC (ES-SCLC) (6–9). Based on these results, the guidelines have updated the recommended initial regimen for ES-SCLC. The FDA has also approved pembrolizumab and nivolumab as third-line regimens for advanced SCLC according to the results of two phase I/II studies (10, 11). Thus, the post-first-line immunotherapy for SCLC is unclear.

Camrelizumab is a PD-1 blocker developed by Hengrui Pharmaceutical. In China, the National Medical Products Administration has approved camrelizumab for use in multiple types of tumors, excluding SCLC. The PASSION study is a randomized controlled trial designed to assess the potential of combining apatinib and camrelizumab as a second-line treatment for ES-SCLC (12). Another historical control clinical study evaluated apatinib plus camrelizumab as a maintenance treatment after chemotherapy plus camrelizumab in untreated ES-SCLC (13). However, the efficacy of camrelizumab for the treatment of SCLC remains unclear. Therefore, SCLC patients were treated with camrelizumab, and the efficacy and safety of camrelizumab has been described in detail in this manuscript.

## 2 Methods

This retrospective case series study was carried out at Beijing Shunyi District Hospital. SCLC patients who received camrelizumab between August 2019 and December 2021 were enrolled in the study. Each patient was diagnosed histopathologically. Patients with incomplete clinical information were excluded. Clinical data of all patients were retrieved from the electronic medical record system. Clinical information included sex, age, clinical stage, metastasis site, line of treatment, and concomitant treatment plan.

The effectiveness of camrelizumab was evaluated based on the Response Evaluation Criteria in Solid Tumors (v.1.1). The methods of follow-up for survival included searching case records and telephonic follow-up. The follow-up period for this study ended on 30 June 2022.

This study was conducted under the guidance of the Declaration of Helsinki. The Beijing Shunyi District Hospital Research Ethics Committee granted ethical approval (Approval Number: 2022-L-012). Written informed consent was obtained from all participants.

### 2.1 Statistical analysis

The patients’ clinical information and efficacy evaluation results are presented by descriptive statistics. The survival data were analyzed by the Kaplan–Meier method. Differences were considered statistically significant at p < 0.05. Statistical analyses and visualization were performed by SPSS v21.0 (IBM) and GraphPad Prism v8.0.2 (GraphPad Software).

## 3 Results

This study enrolled a total of 12 patients. The patients’ ages ranged from 57 to 78 years, with an average age of 66.7 years. Nine patients had ES-SCLC, and three patients presented with recurrence in a limited stage (according to the Veterans Administration Lung Study Group). Of all the patients, 58.3% received second-line or above treatment, and four accepted camrelizumab treatment as a third-line or above. Half of the patients received a combined etoposide regimen. Two patients received anti-angiogenic therapy. Detailed information on patients is displayed in **Table 1**.

**Table 1:** Clinical information and disease characteristics of study cases.

Characteristic	N = 12	Percentage (%)
Gender		
Male	8	66.7
Female	4	33.3
Age (years)		
≤65	5	41.7
> 65	7	58.3
Clinical stage (VALG)		
Limited stage	3	25.0
Extensive stage	9	75.0
Metastasis site		
Lymph nodes	6	50.0
Visceral	6	50.0
Line of treatment		
First line	5	41.7
Second line	3	25.0
≥Third line	4	33.3
Concomitant treatment plan		
Etoposide	6	50.0
Other chemotherapy	4	33.3
Tumor anti-angiogenesis therapy	2	16.7

VALG, Veterans Administration Lung Study Group.

**Figure 1:**
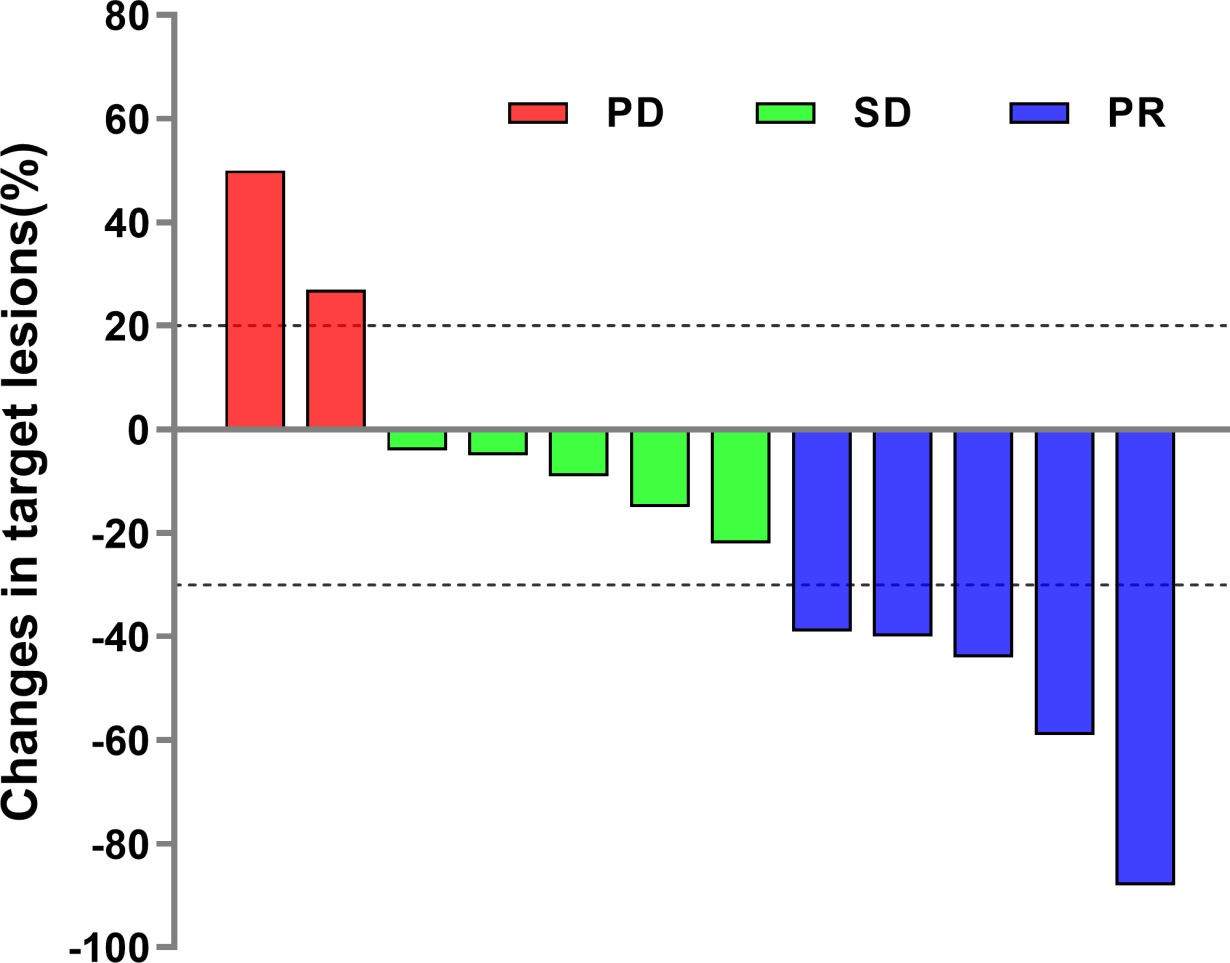
Waterfall plot showing best objective treatment response to camrelizumab for each patient.

The disease control rate (DCR) and objective response rate (ORR) for the 12 SCLC patients were 83.3% (7/12) and 41.7% (5/12), respectively (**Table 2**). None of the patients reached complete response (CR), and five patients who reached partial response (PR) received the combined chemotherapy regimen. The duration of response in one case was 7 months. In the subgroup of patients who received third-line treatment or above, the DCR and ORR were 100% (4/4) and 25% (1/4), respectively. The specific efficacy summary is shown in **Table 3**. The optimal treatment response is shown in **Figure 1**.

**Table 2:** Efficacy evaluation of SCLC patients.

Evaluation criterion	N = 12
CR	0 (0.0%)
PR	5 (41.7%)
SD	5 (41.7%)
PD	2 (16.6%)
**ORR (CR + PR), % 95%(CI)**	41.7% (15.2%–72.3%)
**DCR (CR + PR + SD), % 95%(CI)**	83.3% (51.6%–97.9%)
**Duration of response, month (range)**	4 (1–7)

SCLC, small cell lung cancer; CR, complete response; PR, partial response; SD, stable disease; PD, progressive disease; ORR, overall response rate; DCR, disease control rate.

**Table 3:** The specific efficacy of study patients.

No.	Gender	Age (years)	Clinical stage	Line of treatment	Combination therapy	Clinical effect
1	Male	76	Extensive stage	2	Irinotecan	PR
2	Male	73	Extensive stage	2	Etoposide	PD
3	Male	67	Limited stage	4	Apatinib	SD
4	Female	57	Extensive stage	3	Anlotinib	SD
5	Male	57	Extensive stage	2	Irinotecan	PD
6	Female	63	Extensive stage	4	Docetaxel	SD
7	Female	68	Extensive stage	1	Etoposide	PR
8	Female	63	Extensive stage	3	Paclitaxel	PR
9	Male	59	Limited stage	1	Etoposide	PR
10	Male	70	Extensive stage	1	Etoposide	SD
11	Male	78	Limited stage	1	Etoposide	PR
12	Male	69	Extensive stage	1	Etoposide	SD

PR, partial response; PD, progressive disease; SD, stable disease.

**Figure 2:**
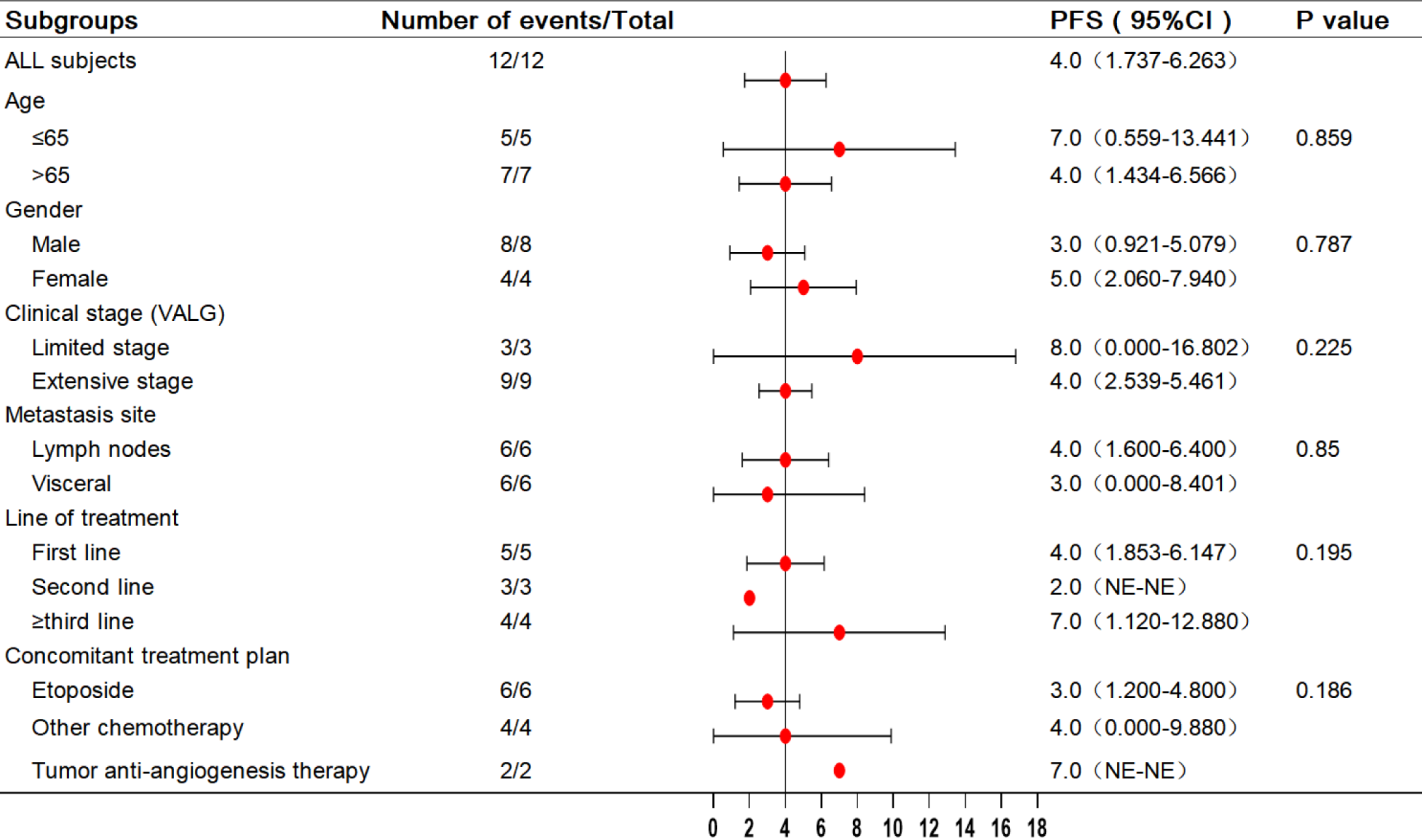
Forest plot of subgroup analysis with PFS in 12 patients. PFS, progression-free survival.

**Figure 3:**
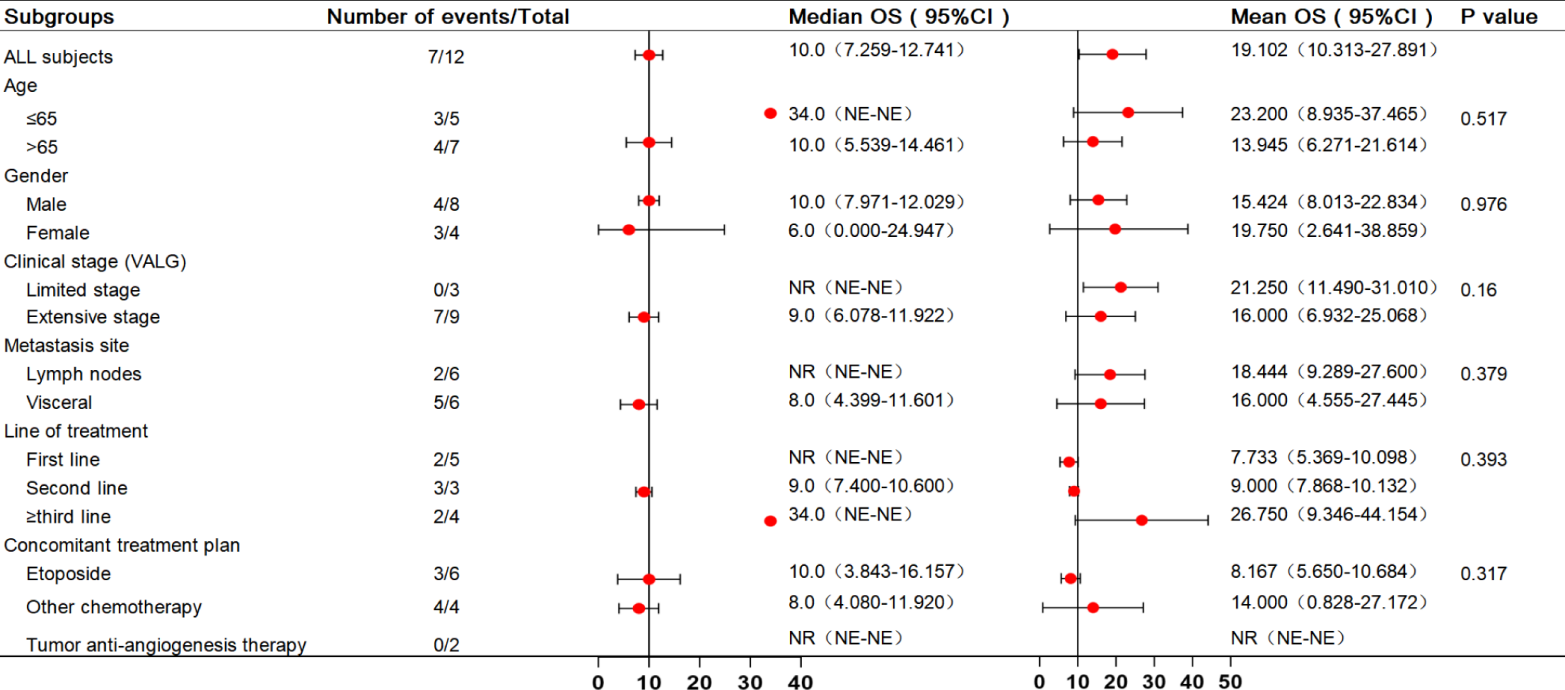
Forest plot of subgroup analysis with OS in 12 patients. OS, overall survival.

In the analysis of all enrolled patients, the median PFS was 4 months (95% confidence interval [CI]: 1.737–6.263 months). In patients with third-line treatment or above, the median PFS reached 7 months (95% CI: 1.12–12.88 months). Additionally, the median PFS with anti-angiogenic drugs was 7 months. All eligible patients’ median and mean overall survival (OS) were 10 and 19.102 months, respectively, and the 1-year survival rate was 25%. Notably, the 1-year survival rate was 75% for patients treated in the third or later-line (range = 5–34 months), with a median OS of 34.0 months, including two patients who received combined oral anti-angiogenic drugs. The analysis results for the other subgroups are shown in **Figures 2**, **3**.

The cohort’s most frequent treatment-related adverse event was hematological toxicity, and one patient developed agranulocytosis (**Table 4**). Moreover, this cohort’s most prevalent immune-related adverse events were immune-associated pneumonia, reactive cutaneous capillary endothelial proliferation (RCCEP), and thyroid dysfunction, as shown in **Table 5**. No grade 4 immune-related toxicities or severe adverse events were reported at the end of the follow-up. One patient experienced an exacerbation of preexisting diabetes and reached grade 3 hyperglycemia.

**Table 4:** Summary of treatment-related adverse events.

Event	N = 12	Percentage (%)
Any adverse event		
Anemia	9	75.0
White blood cell count decreased	8	66.7
Neutrophil count decreased	8	66.7
Platelet count decreased	6	50.0
Hand–foot syndrome	3	25.0
Asthenia	2	16.7
Astriction	2	16.7
Hypertension	2	16.7
Protein urine	2	16.7
Aspartate aminotransferase increased	2	16.7
Blood creatinine increased	1	8.3
Nausea	1	8.3
CTC (common terminology criteria) grade 3 or higher adverse events		
Neutrophil count decreased	6	50.0
White blood cell count decreased	4	33.3
Platelet count decreased	1	8.3
Protein urine	1	8.3

**Table 5:** Summary of immune-related adverse events.

Event	N = 12	Percentage (%)
Any adverse event		
RCCEP	4	33.3
Pneumonitis	4	33.3
Hypothyroidism	1	8.3
Hyperthyroidism	1	8.3
Adrenocortical hypofunction	1	8.3
Hyperglycemia	1	8.3
Rash	1	8.3
CTC grade 3 or higher adverse events		
Hyperglycemia	1	8.3

RCCEP, reactive cutaneous capillary endothelial proliferation. CTC, common terminology criteria.

## 4 Discussion

Presently, ICIs have been approved as a treatment option for SCLC. However, not every patient can receive standard treatment, owing to economic reasons and issues with drug accessibility. Although camrelizumab has not been approved for the treatment of SCLC, the PASSION study has confirmed its potential antitumor activity (12). This case series study presents real-world data on the application of camrelizumab for treating SCLC. These results demonstrate that camrelizumab provides potential benefits and safety for treating SCLC patients. For the 12 patients included in the study, the DCR was 83.3%, ORR was 41.7%, and the median PFS reached 4 months, with a median OS of 10 months. Among them, five patients who reached PR were treated with camrelizumab combined with chemotherapy. In the subgroup analysis, camrelizumab demonstrated favorable results in third-line and above therapies in SCLC. This subgroup exhibited a median PFS of 7 months, a median OS of 34 months, an ORR of 25%, and a DCR of 100%. Furthermore, camrelizumab combined with oral anti-angiogenic drugs demonstrated potential efficacy, as indicated by the median PFS (7 months).

Up to now, the survival data of SCLC patients receiving third-line treatments have not been optimistic. Nivolumab is the first ICI approved for the third or post-third-line treatment in SCLC according to CheckMate 032 (10). With updated results, the median OS of 147 patients with nivolumab was 5.7 months (95% CI: 3.8–7.6 months) (14). The CheckMate 331 study compared nivolumab with chemotherapy as a second-line regimen in progressed SCLC patients after standard chemotherapy (15). This study demonstrated no significant improvement in the nivolumab group vs. the chemotherapy group (median OS [7.5 vs. 8.4 months] and median PFS [1.4 vs. 3.8 months]). However, the results of the Chinese cohort showed that the median OS of the nivolumab group was slightly longer than that of the chemotherapy group (11.5 vs. 7.0 months; hazard ratio [HR] = 0.70; 95% CI: 0.42–1.17). Additionally, two clinical studies evaluated the efficacy of pembrolizumab in treating SCLC. The phase 1b KEYNOTE-028 trial included 24 recurrent or metastatic SCLC patients (16). In the SCLC cohort, the ORR was 33.3%, and the median OS was 9.7 months (95% CI: 4.1–Not Reached). KEYNOTE-158 was a phase 2 study that included 107 SCLC patients who relapsed after treatment (17), the ORR was 18.7%, and the median OS was 9.1 months (95% CI: 5.7–14.6 months). A pooled analysis result of both the studies, KEYNOTE-158 and KEYNOTE-028, showed a median OS with two or more lines of treatment of 7.7 months (95% CI: 5.2–10.1 months) for SCLC (11). The results of our study are equivalent to the aforementioned clinical trials.

ICIs are a promising treatment for SCLC; however, some patients still develop drug resistance. Cancer cells, via multiple molecular mechanisms, induced an immunosuppressive and negative regulation of cytotoxic T cells (18). SCLC has a complex tumor microenvironment (TME) that regulates the PD-1/PD-L1 pathway and allows cancer cells to escape immune surveillance (19). ICIs combined with chemotherapy may alter these escape pathways and restore the anti-tumor activity of the immune system. A pooled meta-analysis of four clinical trials, IMpower133, CASPIAN, KEYNOTE604, and ECOG-ACRIN EA5161, found that ICIs combined with chemotherapy significantly improved the ORR, PFS, and OS of SCLC (p < 0.05) (20). Furthermore, the results of ASTRUM-005, an international phase 3 clinical trial, proved that chemotherapy plus serplulimab significantly ameliorated the median OS compared with chemotherapy alone among initial untreated ES-SCLC patients (15.4 vs. 10.9 months, p < 0.001) (8). Another phase 3 multicenter clinical study, CAPSTONE-1, conducted in the Chinese population also confirmed that PD-L1 blocker combined with chemotherapy is superior to chemotherapy in improving median OS in initial untreated ES-SCLC patients (15.3 versus 12.8 months, p = 0.0017) (9). In the present study, five patients who achieved a PR were treated with camrelizumab combined with chemotherapy.

Angiogenesis plays a significant role in establishing and maintaining the TME and is a critical factor in immune escape. Abnormal angiogenesis induces immunosuppression by suppressing antigen presentation, promoting the expression of inhibitory receptors on T cells, and inducing hypoxia (21, 22). In turn, the immunosuppressive environment can promote abnormal angiogenesis. This results in a vicious circle of immunosuppression. Therefore, combining anti-angiogenic drugs with ICIs may be a practical strategy to reverse immunosuppression. Basic research has confirmed that inhibiting the vascular endothelial growth factor (VEGF) pathway can overcome drug resistance in the PD-1/PD-L1 axis (23). Currently, clinical trials have been carried out to evaluate anti-VEGF therapy and combination treatment with anti-VEGF and anti-PD-1 for SCLC. The Eastern Cooperative Oncology Group (ECOG) 3501 trial evaluated the effectiveness of a triple-drug combination involving bevacizumab, etoposide, and cisplatin in treating 63 patients with ES-SCLC (24), in which the median PFS and OS were reported as 4.7 (95% CI: 4.3–5.5) and 10.9 (95% CI: 7.9–12.2) months, respectively. Another phase II single-arm study, CALGB 30306, evaluated a regimen of bevacizumab plus cisplatin and irinotecan for ES-SCLC (25). The survival data were slightly higher than those of irinotecan-based trials. Based on the above data, two phase III clinical studies, IFCT-0802 and GOIRC-AIFA FARM6PMFJM, were conducted to evaluate bevacizumab plus chemotherapy compared with chemotherapy alone (26, 27). Unfortunately, in these studies, neither median PFS nor median OS was significantly prolonged in the bevacizumab plus chemotherapy group. Unlike bevacizumab, ramucirumab specifically targets vascular endothelial growth factor receptor 2 (VEGFR2). In Japan, a recent phase Ib study showed satisfactory efficacy and tolerance to ramucirumab plus irinotecan and cisplatin in the treatment of ES-SCLC (28). We look forward to further randomized controlled trials about ramucirumab in SCLC. Similarly, tyrosine kinase inhibitors (TKIs) targeting VEGFR have shown good results in the treatment of SCLC. Based on the ALTER1202 study, anlotinib has become the standard regimen for treating third-line SCLC in China (29). In the study, anlotinib presented a median PFS of 4.1 compared to 0.7 months for the placebo group (HR: 0.19; 95% CI: 0.12–0.32). Additionally, the median OS for anlotinib was 7.3 compared to 4.9 months for the placebo group (HR: 0.53; 95% CI: 0.34–0.81). Currently, a phase II study (NCT04363255) is being conducted to assess anlotinib in combination with a PD-1 inhibitor as a maintenance therapy for SCLC following first-line treatment. The final results of the study have not yet been announced. Real-world research offers a reference for SCLC treatment. In a retrospective study, as a second- or later-line treatment for recurrent SCLC, the median PFS of anlotinib combined with a PD-1 inhibitor was significantly longer than that of mono PD-1 inhibitor (5.0 vs. 3.0 months; p = 0.005) (30). A prospective single-arm multicenter study evaluated apatinib (an oral TKI that selectively targets VEGFR2) as a first-line regimen for relapsed SCLC patients (31). In the study, apatinib showed a DCR of 79.6%, ORR of 14.3%, and median PFS and OS of 5.6 (95% CI: 3.3–8.0) and 11.2 (95% CI: 7.5–24.0) months, respectively. Another study evaluated apatinib as a third-line or more-line regimen in ES-SCLC, reporting an ORR of 13.6% and a DCR of 95.5% (32). The PASSION study assessed the feasibility of camrelizumab in combination with apatinib as a second-line therapy for ES-SCLC (12). The ORR of 47 patients was 34.0% (95% CI: 20.9−49.3), with the median PFS and median OS of 3.6 and 8.4 months, respectively. In a non-randomized clinical study, 19 patients received standard chemotherapy and camrelizumab followed by apatinib plus camrelizumab as maintenance therapy in untreated ES-SCLC. The study showed the ORR was 89.6%, and the median PFS was 10.25 months (95% CI: 9.40–not reached) (13). The above research provides evidence-based medicine of anti-VEGF plus anti-PD-1 therapy in SCLC. In the present study, patient 3 was treated with camrelizumab combined with apatinib as a fourth-line therapy. The target lesion size was reduced by 22%, and the PFS was 17 months. The patient started taking the drug in March 2020 and was followed up for 27 months. Currently, the patient remains alive. Furthermore, patient 4 was treated with camrelizumab plus anlotinib as a third-line treatment, which resulted in a PFS and OS of 7 and 17 months, respectively.

According to a CAMEL study on NSCLC, the most common non-hematological immune-related toxicity of camrelizumab is RCCEP, observed in 78% of cases, of which grades 1–2 account for 77% (33). Similarly, in our study, RCCEP and pneumonia were the two most common immune-related adverse events (44.4%). Our study showed an acceptable safety profile; only one patient experienced an exacerbation of preexisting diabetes and reached grade 3 hyperglycemia. Interestingly, studies have confirmed that the occurrence of RCCEP predicts camrelizumab efficacy (34). In our study, patient 6 had grade 2 RCCEP; the PFS and OS of this patient were 10 and 34 months respectively. Furthermore, several studies have confirmed that camrelizumab plus anti-angiogenic therapy can significantly lower the occurrence of RCCEP. Indeed, the two patients who received combined anti-VEGFR2 therapy did not develop RCCEP.

The boundedness of this study included its retrospective design and the insufficient sample size to evaluate camrelizumab in SCLC, which might have introduced a bias in the results of the subgroup analysis. Additionally, the present standard third-line regimen for the treatment of SCLC is a monodrug therapy with anlotinib or pembrolizumab/nivolumab; however, this study did not include data on monodrug therapy with camrelizumab. Although the current data on camrelizumab may not alter the standard treatment regimen of ES-SCLC, camrelizumab plus chemotherapy could be a choice in recurrent patients with good status. When the ECOG score is greater than 2, camrelizumab monotherapy or combination with anti-angiogenesis therapy may be a viable option in the real world.

## 5 Conclusions

This retrospective study showed an acceptable efficacy and safety profile of camrelizumab plus chemotherapy or anti-VEGFR2 therapy as a third-line or post-third-line regimen for ES-SCLC. The results suggest that camrelizumab may be a potential regimen option for this population. Nevertheless, we must acknowledge the limitations of our conclusion due to the retrospective nature and small sample size. Therefore, prospective randomized controlled studies with a large sample are needed to confirm our findings.

## Data availability statement

The original contributions presented in the study are included in the article/supplementary material. Further inquiries can be directed to the corresponding author.

## Ethics statement

The studies involving human participants were reviewed and approved by Beijing Shunyi District Hospital Research Ethics Committee. The patients/participants provided their written informed consent to participate in this study. Written informed consent was obtained from the individual(s) for the publication of any potentially identifiable images or data included in this article.

## Author contributions

JT drafted the article, substantially contributed to the conception and design, and finally approved the version to be published. JT, LS, and HW collected data or analyzed and interpreted data. XC gave the final approval of the version to be published. All authors contributed to the article and approved the submitted version.
